# The converging influence of social, economic and psychological factors on public responsiveness to the COVID-19 pandemic in South Africa

**DOI:** 10.1186/s12889-022-13845-y

**Published:** 2022-07-30

**Authors:** Lu-Anne Swart, Naiema Taliep, Ghouwa Ismail, Ashley van Niekerk

**Affiliations:** 1 Institute for Social and Health Sciences, University of South Africa, Johannesburg, South Africa; 2 Masculinity and Health Research Unit, South African Medical Research Council and University of South Africa, Cape Town, South Africa

**Keywords:** COVID-19, South Africa, Adoption of protective behaviours, Adherence to restrictions

## Abstract

**Background:**

This study assessed the influence of social, economic, and psychological factors on South African’s responsiveness to the COVID-19 pandemic. Although the South African government responded quickly to manage the pandemic, the strict lockdown placed a significant burden on the population. Understanding the converging influence of social, economic, and psychological factors on the population’s responsiveness is important for improving people’s cooperation in controlling COVID-19 and for supporting individuals and communities during the ongoing and future pandemics.

**Methods:**

Using data collected from a national telephonic survey (December 2020 to March 2021), we assessed whether selected social, economic and psychological factors were related to: 1) adoption of COVID-19 behavioural measures (hand hygiene, wearing of face masks, and physical distancing), and 2) adherence to government restrictions on movement.

**Results:**

South Africans were highly responsive to the pandemic with respondents generally reporting that they very often engaged in the protective behaviours and often to very often adhered to government restriction on movement. However, those from the white population group; with a higher education; living in uncrowded households; who perceived less vulnerability to contracting COVID-19; supported the measures; trusted the scientists; thought the measures by government were implemented fairly and fairly enforced by the police; felt more anxious, sad, hopeless, isolated, angry or had trouble sleeping; inclined to engage in coping behaviour, were more likely to adopt COVID-19 protective behaviours. Furthermore, females, those with a lower education, those less likely to have experienced poverty since the beginning of lockdown; who perceived greater vulnerability to COVID-19, trusted government, and were more supportive of the behavioural measures were more likely to adhere to the restrictions of movement.

**Conclusions:**

Strengthening the South African population’s responsiveness to the pandemic requires supporting those living in poor socioeconomic circumstances, promoting trust in the scientific evidence, and ensuring that the measures by government are perceived to be fairly implemented and fairly enforced by the police. Due to the impact on livelihoods, restrictions of movement should only be considered if necessary, and this will require trust and confidence in government and strategies to support those experiencing financial hardship.

## Background

The South African government responded quickly to contain the spread and ‘flatten the curve’ of COVID-19 in the country. With the first case reported on the 5th March 2020, government declared a national state of disaster on 15th March, which was followed by a national lockdown on 27th March, with government later announcing a phased approach that would manage lockdown restrictions over five stages [1–4]. The impact of the broader lockdown measures, specifically the restrictions on movement and economic activity, placed a significant burden on individuals, families, and communities. In January 2022, the country emerged from a fourth wave of the pandemic with COVID-19 infection rates at around 3 590 399 cases and deaths at around 94 491 [5]. While the more restrictive lockdown measures, such as curfews and partial closure of borders and the economy (e.g., nightclubs and sales of alcohol) have been lifted, restrictions on gatherings and mandatory wearing of facemasks in public remained in place. South Africa also began its vaccination rollout programme in February 2021, with approximately 25% of the population fully vaccinated by January 2022 [5]. However, vaccination alone is insufficient to curb the outbreak of COVID-19, and at least while the vaccine is being distributed to the population, non-therapeutic interventions (hand hygiene, physical distancing, using facemasks in public, avoiding gathering and limiting non-essential movement outside the home) have been noted as key to mitigating a resurgence of the disease [6, 7]. Against this background, there is an urgent need to examine the responsiveness of the South African population to the State ascribed COVID-19 preventive behaviours. This may help to identify persons at increased risk of non-adherence, and to locate entry points into improving people’s cooperation in controlling COVID-19. Such information could be used to strengthen vulnerable communities during and beyond precarious moments in history.

A rapidly growing body of research has identified a range of mutually influencing social, economic, and psychological factors that affect individuals’ adoption of COVID-19 protective behaviours. Socioeconomic variables including demographics (age, gender, population group), education, and income level appear to influence the adoption of COVID-19 preventive behaviours. For example, studies from Korea, India, and Indonesia suggest that elderly populations, women, and those with a higher education and a stable income are more likely to engage in COVID-19 protective behaviours [8–10]. Economic factors have a marked influence on health behaviour and health outcomes by restricting access to health care, the appropriate resources and environmental conditions, and by increasing stress [11, 12]. In South Africa, a substantial proportion of the country’s population live in conditions of poverty; in neighbourhoods marked by a lack of infrastructure, including inadequate housing and sanitisation, and high levels of overcrowding. The standard public health protective measures, such as hand washing and physical distancing are difficult to implement in informal settlements and other working class neighbourhoods with high population densities and inadequate access to water and sanitation [13, 14]. Furthermore, lockdown restrictions in South Africa have also had a financial impact on many individuals with the closing down of businesses and increasing unemployment. Thus, the number of people in the country living in poverty has increased since the pandemic, with most of those who were already poor having had their material circumstances further impoverished [15]. It is, therefore, important to consider the influence of economic factors on the adoption of COVID-19 preventive behaviours.

Psychological factors also appear to play a significant role in the adoption of COVID-19 preventive behaviours. Studies have drawn on the Health Belief Model or Protection Motivation Theory to understanding the adoption of COVID-19 preventive behaviours [16–18]. According to these models, individuals are more likely to engage in preventative behaviour if they perceive that they are at risk (perceived vulnerability) or if they perceive that the recommended behaviours will effectively minimise that risk (i.e. response efficacy beliefs). A study of participants from 70 countries, and other studies conducted in Iran and Ethiopia have shown support for these models, and suggest that perceived vulnerability and response efficacy are positively associated with the adoption of COVID-19 protective behaviours [16–18].

The science on COVID-19 is constantly evolving, and this has contributed to the rapid change of information, mixed messages, and inconsistencies in recommendations [19], all of which could undermine public trust in the sources of information and ultimately influence their adherence to the preventive measures. Research across several countries suggests that trust in COVID-19-related information provided by government and scientists is significantly associated with adherence to COVID-19 protective measures [20–24]. Another study in Finland also found that individuals who hold conspiratorial beliefs and lower trust in information sources were less likely to have a positive response to COVID-19 [19]. Perceptions of the fairness of measures taken by government and fairness in the enforcement of the measures [25] may also be linked to the adoption of behaviours. In South Africa, state corruption as well as decades of economic austerity that have resulted in widening inequality and poverty have resulted in demonstrably low trust in government among much of the population [26]. With respect to fairness of COVID-19 measures, as with most countries, many of the country’s working-class population could not work from home because they did not have the resources or their type of work did not allow them to do so. Moreover, there was a noted spike in the South African Police Service forcibly implementing COVID-19 protocols among the country’s poorer population [27].

The restriction on movement and economic activity facilitated by the COVID-19 lockdowns have also had a psychological impact with respect to increases in stress, anxiety, depression, loneliness, and an uncertainty about the future [28–31]. In South Africa, the loss of employment and food insecurity has had dire psychological effects, with more than half of the country’s population having experienced mental distress at least once since the first lockdown [29, 32]. Depression and stress have been associated with lack of adherence to medical treatments [33–35] and may also be associated with the adoption of COVID-19 protective behaviours. Studies in Japan suggest that anxiety, depression and loneliness were associated with lower levels of engagement in COVID-19 preventive behaviours [36, 37]. However, a longitudinal study in the UK found no evidence that worries about the future and loneliness were related to one’s compliance with pandemic related restrictions [38]. Adaptability and coping strategies also appear to be important factors, with a study among participants from Australia, US, UK, and Canada showing that compared to individuals who adhered to the protective behaviours, those who did not adhere were less likely to have used coping strategies adaptive for COVID-19 (self-distraction, active coping, planning) and more likely to have used maladaptive coping strategies (denial, substance use, behavioural disengagement) [39].

The present study examines the influence of key social, economic, and psychological factors on South African’s responsiveness to the COVID-19 pandemic. The study objectives were to determine whether social factors (age, gender, population group, education, number of household members), financial impact of COVID-19, perceived vulnerability, support for mitigation measures, trust in government and scientists and views on procedural justice, as well as the psychological impact of the COVID-19 lockdown were related to: 1) adoption of COVID-19 behavioural measures (hand hygiene, wearing of face masks, and physical distancing), and 2) adherence to government restrictions on movement. We focus on restrictions on movement as these were specifically linked to the constraints placed on economic activity and sustainable livelihoods.

## Methods

### Participants and procedures

This study is part of a larger project on the related social, economic and psychological impact of the COVID-19 pandemic in South Africa. The Bureau of Market Research (BMR) was commissioned to conduct the survey through Computer-Aided Telephonic Interviews (CATI) from December 2020 to March 2021. This period coincided with the second wave of the COVID-19 pandemic in South Africa when lockdown measures were more restrictive. As face-to-face interviews were not possible at the time, CATI was considered as the most appropriate method to collect data. A proportionate stratified sample of 2 400 adults (aged 18 years and older) was designed using the number of households in each province as the weighting factor. To sufficiently cover the towns and villages representing urban and rural areas in the different provinces, the relative sample sizes at provincial level were adjusted to ensure that no sub-cluster population was below 30 respondents. Adults were randomly selected from telephone directories to participate in the study. After telephone contact had been established, each respondent provided verbal informed consent before proceeding with the interview. All telephone interviewers were trained on questionnaire administration and were supervised by BMR.

The survey questionnaire was developed by the research team to collect information on economic status and living circumstances, perceptions of and responsiveness to COVID-19 and protective measures and restrictions, views on the government’s handling of the pandemic, and the financial and psychological impact of the pandemic. The instrument was evaluated internally by six researchers for face validity and piloted by BMR among 20 adults residing in the province of Gauteng to assess the feasibility of the questionnaire. The study received ethical approval from the University of South Africa’s College of Human Sciences and the BMR research ethics committees.

Approximately 2619 adults were approached, with 2118 agreeing to participate in the study, thus yielding a response rate of 80.8%. Following the removal of cases with missing values for the variables under investigation, the final sample for our analysis comprised 1615 respondents.

For multiple linear regression incorporating 16 explanatory variables with 80% power and *a* error probability of 5%, it is estimated that a sample of 1 000 will detect a *R*^2^ values of 2 percent and above [40] And therefore the final sample size of 1615 was considered to be adequate for the purposes of our analysis.

### Variables and measures

The dependent variables in our study are 1) adoption of protective behaviours and 2) adherence to restrictions. Adoption of protective behaviours was measured using the mean of three items asking respondents how often they had engaged in COVID-19 protective behaviours, including handwashing, wearing a face mask in public, and social distancing when outside their household, Scores were *never* (1) *just once or twice* (2) *often* (3) and *very often* (4) (Cronbach’s α = 0.76). A 3-item subscale assessed adherence to government restrictions on movement, including visiting family, friends, or neighbours, leaving home for non-essential activities, and attending gatherings of more than 10 people, which were combined into an index of adherence to restrictions (Cronbach’s α = 0.58).

Based on our review of the literature, 16 social, economic and psychological explanatory variables were selected from the survey data. The social and economic variables for the study included demographics and the financial impact of lockdown. The *demographic variables* comprised of age, gender, race, education, and number of household members. Respondents’ age was measured in 5-year age groups from *18–19 years* (1), *20–24 years* (2), *25–29 years* (3), *30–34 years* (4), *35–39 years* (5), *40–44 years* (6), *45–49 years* (7), *50–54 years* (8), *55–59 years* (9), *60–64 years* (10), to *65 years and older* (11) to align to routine descriptive statistical conventions [41]. Gender^1^ was coded as *male* (1) and *female* (2). Race^2^ was coded as 1 if the respondent identified as black (black, coloured or Indian) and 2 if white. Education comprises of the educational level attained by the respondent and ranges from *no formal education* (1) to *postgraduate degree* (9). The variable number of household members is the actual number of members living in the respondent’s household. Two variables captured the *financial impact of lockdown*: experience of poverty and loss of income. Poverty was measured using an adapted version of the Afrobarometer Lived Poverty Index [42] and comprised the mean of six items: how often respondents or anyone in their household had gone without food, water, medicines or medical treatment, cash income, electricity, and shelter since the start of lockdown. Scores on the index ranged from *never* (1), *just once or twice* (2), *several times* (3), *many times* (4), to *always* (5) (Cronbach’s α = 0.64). Loss of income, based on a 5-point scale item (0%, 25%, 50%, 75%, or 100%), asked how much income of a respondent’s household earned before lockdown, was lost since the beginning of lockdown.

The psychological variables were grouped as: vulnerability to COVID-19, importance of the measures for curbing COVID-19, trust in information about COVID-19 from government and scientists, procedural fairness of the measures to curb COVID-19, and psychosocial wellbeing. *Vulnerability to COVID-19* was measured using the mean of two items asking respondents how concerned they were that they could become infected with COVID-19 at some time and how concerned they were that someone in their family could become infected with COVID-19. The items were assessed on a 5-point scale ranging from *not at all* (1) to *extremely* (5).

Two variables were used to capture *support for the measures for curbing COVID-19:* importance of protective behaviours and importance of restrictions on movement. Perceptions of the importance of protective behaviours were measured using the mean of four items asking respondents how they rate the importance of handwashing or sanitising, wearing a face mask in public, distancing from people in public spaces, and staying at home to curb the spread of the virus. Each item was assessed on a 5-point scale ranging from *not important at all* (1) to *very important* (5) (Cronbach’s α = 0.82). Respondents’ perceptions on the importance of restrictions on movement consisted of the mean of four items assessing how respondents rated the importance of lockdown restrictions, such as no visiting of family, friends and neighbours; the closing of schools; closing of sectors of the economy; and the closing of religious institutions (Cronbach’s α = 0.69).

*Trust in COVID-19 information* consisted of two variables: information from government and scientists. Trust in information from government was measured using a single item asking respondents how much they trust the information from government about COVID-19. Responses were measured on a 4-point scale ranging from *not at all* (1) to *completely trust* (4). Trust information from scientists, based on a 4-point scale item, asked respondents how much they trust the information from scientists and researchers about the coronavirus. The responses ranged from *not at all* (1) to *completely trust* (4).

*Perceived procedural fairness* of the COVID-19 lockdown measures included two variables: Fairness of measures taken by government and fairness in the enforcement of the measures. Fairness of measures taken by government consisted of the mean of two items that asked respondents to indicate whether they agreed the measures taken by government were for the benefit of all South Africans and whether the measures were necessary to protect all lives. The items were assessed on a 5-point scale ranging from *strongly disagree* (1) to *strongly agree* (5). Fairness in the enforcement of the measures was assessed using the mean of three items asking respondents whether the South African Police Service (SAPS) or the South African Army treated everyone equally and fairly; whether SAPS or the South African Army were too harsh on people for not obeying the regulations; and if the respondent was aware of someone who was treated harshly by SAPS or the South African Army (Cronbach’s α = 0.58).

The *psychological impact of COVID-19* consisted of two variables: emotional wellbeing and coping behaviour. Emotional wellbeing was assessed by the mean of six items asking respondents how often they felt anxious, sad, hopeless, isolated, angry, and had trouble sleeping in the last seven days. Scores on the index ranged from *rarely or none of the time (less than 1 day)* (1) to *most or all of the time (5–7 days)* (3) (Cronbach’s α = 0.85). Coping behaviour consisted of the mean of four items, that asked respondents to indicate how much, compared to before lockdown, they had engaged in a pleasant activity not work related; had a good time with their family engaged in some form of meditation or prayer; and engaged in some form of physical activity. Scores for the items ranged from *much less* (1) to *much more* (5) (Cronbach’s α = 0.61).

### Data analysis

Descriptive statistics were computed to describe the study variables. We used multivariate linear regression models to examine the associations between the explanatory variables and the dependent variables (adoption of protective behaviours; adherence to restrictions on movement). Preliminary analyses were conducted to determine whether the data met the assumptions of regression, namely outliers, normality, linearity, homoscedasticity and multicollinearity. Significance level for all analyses was set at *p* = 0.05. All descriptive, correlational, and regression analyses were performed using IBM SPSS Statistics for Windows, version 26 (IBM Corp., Armonk, N.Y., USA).

## Results

The social, economic and psychological characteristics (explanatory variables) of the 1 615 study participants are described in Table 1. Respondents were on average aged between 40–44 years, most (67.7%) were female, black (68.2%), with an average education level consisting of a post-secondary qualification other than a university degree and lived in households with an average of 4 members. With regard to the financial impact of lockdown, the mean score for the experience of the poverty index was 1.41 indicating than on average respondents or their family members had never or just once or twice gone without food, water, medicines or medical treatment, cash income, electricity, and/or shelter since the start of lockdown. However, respondents reported that on average around 25% of their household income earned before lockdown began was lost since the start of lockdown.

**Table 1:** Participants’ characteristics: explanatory variables

Variable	Mean/%	*SD*	Scale
**Socioeconomic**			
Age	6.69	3.109	1–11
Gender (Female)	67.7%		
Population group (Black)	68.2%		
Education	6.46	1.832	1–9
Number of household members	4.06	2.844	-
**Financial impact of lockdown**			
Experience of poverty	1.41	0.514	1–5
Loss of income	2.14	1.347	1–5
**Perceived vulnerability to COVID-19**	3.74	1.15	1–5
**Support for measures to curb COVID-19**			
Importance of behavioural measures	4.73	0.519	1–5
Importance of restrictions on movement	3.13	0.990	1–5
**Trust government and institutions**			
Trust information from government on coronavirus	2.63	1.024	1–4
Trust information from scientists	2.86	0.946	1–4
**Procedural justice**			
Fairness of measures taken by government	3.74	1.029	1–5
Fairness of enforcement of measures	2.97	0.959	1–5
**Psychosocial impact**			
Emotional distress	1.58	0.561	1–3
Coping behaviour	3.41	0.953	1–5

In general, perceived vulnerability was high (x̄ = 3.74) with respondents very much concerned that they or their family would become infected. Respondents also tended to perceive that the protective behaviours (hand hygiene, face mask, keeping a safe distance, and staying at home) were very important (x̄ = 4.73) to curb the spread of COVID-19, and regarded the restrictions on movement (no visiting of family, friends and neighbours, and the closing of schools, sectors of the economy, and religious institutions) as moderately important (x̄ = 3.13).

Overall, respondents mostly trusted the information on COVID-19 from government (x̄ = 2.63) and scientists (x̄ = 2.86). Although respondents tended to agree that the measures taken by government were procedurally fair (x̄ = 3.74), respondents were on average neutral (neither agreeing of disagreeing) (x̄ = 2.97) regarding the perceived fairness of the enforcement of the measures.

With a mean score of 1.58 for emotional wellbeing, respondents on average reported feeling anxious, sad, hopeless, isolated, angry, and/or having trouble sleeping occasionally or a moderate amount of time in the past seven days. With respect to coping behaviours, respondents reported generally that they had engaged in certain forms of activities about the same to somewhat more than before lockdown (x̄ = 3.41).

Table 2 displays the descriptive statistics for the outcome variables of the study, namely adoption of protective behaviours (x̄ = 3.74) and adherence to government restrictions on movement (x̄ = 3.44). Adoption of protective behaviours was very high, with respondents reporting on average that they very often washed hands (x̄ = 3.76), wore a mask in public (x̄ = 3.80), and kept a safe distance from people outside the household (x̄ = 3.66). Similarly, adherence to government restrictions on movement was also high with respondents on average indicating that they never or once/twice visited or had family, friends or neighbours visit (x̄ = 3.28), left their home for non-essential activities (x̄ = 3.36), and attended a gathering of more than 10 people (x̄ = 3.67).

**Table 2:** Descriptive statistics on outcome measures: responsiveness to COVID-19 measures

Item	Mean	*SD*	Scale
In the past 7 days how often have you: (Never, once/twice, often, very often)			
**Adoption of protective behaviours**			
Washed hands with soap for 20 s (or use hand sanitiser)	3.76	.505	1–4
Worn a face mask when in public	3.80	.448	1–4
Kept a safe distance from people outside of my household	3.66	.624	1–4
Overall measure	**3.74**	.436	1–4
**Adherence to government restrictions on movement**			
Visited family, friends or neighbours or had family, friends or neighbours visit your household^a^	3.28	.858	1–4
Left your home not for essential activities (e.g., grocery shopping, medical appointments, work)^a^	3.36	.849	1–4
Attended a gathering where there were more than 10 people (e.g. wedding, birthday party, funeral or a religious service)^a^	3.67	.649	1–4
Overall measure	**3.44**	.584	1–4

*Note*. ^a^ reverse scored

Table 3 displays the multivariate linear regression results for the association between the social, economic, and psychological explanatory variables and 1) adoption of preventive behaviours and 2) adherence to government restrictions on movement. Of the social variables, population group, education and number of household members were significantly related to the adoption of preventive behaviours. White (compared to black) (*β* = -0.083, *p* = 0.002), those with higher education (*β* = 0.105, *p* = 0.000), and those living in households with fewer members (*β* = -0.058, *p* = 0.012), were more like to adopt the behavioural measures. The socioeconomic variables, age, gender and the financial impact of lockdown, including loss of income and the experience of poverty were unrelated to the adoption of protective behaviours.

**Table 3:** Multiple linear regression of explanatory variables on responsiveness to COVID-19 measures

		**Behavioural measures**					**Restrictions on movement**			
**Explanatory variables**	**B (95% CI)**	***SE***	**Beta**	***t***	***p***	**B (95% CI)**	***SE***	**Beta**	***t***	***p***
**Constant**	1.869 (1.621 – 2.116)	.126		14.790	.000	3.285 (2.919 – 3.652)	.187		17.601	.000
**Socioeconomic**										
Age	.005 (-.002 – .012)	.004	.035	1.397	.163	.009 (-.001 – .019)	.005	.049	1.747	.081
Gender	.011 (-.029 – .052)	.021	.012	.544	.587	-.150 (-.210 – -.090)	.031	-.120	-4.916	.000
Population group (black vs white)	-.077 (-126 – -.029)	.025	-.083	-3.129	.002	.053 (-.019 – .125)	.037	.042	1.448	.148
Education	.025 (.014 – .035)	.005	.105	4.656	.000	-.052 (-.067 – -.036)	.008	-.163	-6.551	.000
Number of household members	-.009 (-.016 – -.002)	.004	-.058	-2.505	.012	-.007 (-.017 – .004)	.005	-.032	-1.275	.203
**Financial impact of lockdown**										
Experience of poverty	-.020 (-.061 – .021)	.021	-.023	-.952	.341	-.110 (-.170 – -.050)	.031	-.097	-3.585	.000
Loss of income	.005 (-.010 – .019)	.007	.014	.622	.534	-.005 (-.027 – .017)	.011	-.011	-.431	.667
**Perceived vulnerability to COVID-19**	-.028 (-.045 – -.011)	.009	-.073	-3.208	.001	.030 (.005 – .055)	.013	.059	2.356	.019
**Support for measures to curb COVID-19**										
Importance of behavioural measures	.318 (.277 – .358)	.021	.376	15.285	.000	.124 (.064 – .185)	.031	.110	4.049	.000
Importance of restrictions on movement	.047 (.026 – .068)	.011	.106	4.429	.000	.014 (-.16 – .045)	.016	.024	.910	.363
**Trust government and institutions**										
Trust information from government on coronavirus	-.007 (-.032 – .018)	.013	-.017	-.569	.569	.047 (.010 – .084)	.019	.083	2.483	.013
Trust information from scientists	.029 (.003 – .054)	.013	.062	2.193	.028	-.024 (-.062 – .014)	.019	-.039	-1.232	.218
**Procedural justice**										
Fairness of measures taken by government	.027 (.005 – .049)	.011	.063	2.394	.017	-.012 (-.045 – .020)	.017	-.021	-.732	.464
Fairness of enforcement of measures	-.027 (-.048 – -.007)	.011	-.060	-2.603	.009	-.008 (-.039 – .022)	.016	-.014	-.529	.597
**Psychosocial wellbeing**										
Emotional distress	.063 (.027 – .099)	.018	.081	3.424	.001	-.034 (-.088 – .019)	.027	-.033	-1.249	.212
Coping behaviour	.035 (.015 – .055)	.010	.076	3.430	.001	.014 (-.015 – .044)	.015	.024	.963	.336

*R*^2^ = .259; *F* = 34.871 *p* = .000

*R*^2^ = .098; *F* = 10.889 *p* = .000

With the exception of trust in the information on COVID-19 provided by government, all of the psychosocial variables were significantly associated with the adoption of protective behaviours. Respondents who supported the importance of the behavioural measures (*β* = 0.376, *p* = 0.000) and restrictions on movement (*β* = 0.106, *p* = 0.000), were more trusting of the information provided by scientists (*β* = 0.062, *p* = 0.028), were more agreeable that the government measures were fair and reasonable (*β* = 0.063, *p* = 0.017), but less agreeable with fairness of the enforcement of the measures (*β* = -0.027, *p* = 0.009), experienced greater emotional distress (*β* = 0.081, *p* = 0.001), and were more likely to engage in coping behaviour (*β* = 0.076, *p* = 0.001) and to adopt the protective behaviours.

With respect to the adherence to restriction on movement, the results show a somewhat different pattern. Among the social and financial impact variables, gender, education, and the experience of poverty were significantly related to the adherence of the restrictions while age, population group, number of household members and loss of income were not related. Respondents who were female (*β* = -0.120, *p* = 0.000), those with lower educational attainment (*β* = -0.163, *p* = 0.000), and less likely to experience poverty (*β* = -0.097, *p* = 0.000) were more likely to adhere to the restrictions.

Perceptions of the importance of the behavioural measures and trust in the information from government were the only psychological variables that were significantly associated with adherence to the restrictions on movement. Respondents who perceived the behavioural measures to be of more importance (*β* = 0.110, *p* = 0.000) and were more trusting of the information provided by government (*β* = 0.083, *p* = 0.000) were more likely to adopt the protective behaviours.

In summary, the results of the study indicate that South Africans were in general highly responsive to the pandemic. Those from the white population group; with a higher education; living in uncrowded households; who perceived less vulnerability to contracting COVID-19; supported the behavioural measures and restrictions; trusted the scientists; thought the measures by government were implemented fairly and fairly enforced by the police; felt more anxious, sad, hopeless, isolated, angry or had trouble sleeping; inclined to engage in coping behaviour, were more likely to adopt COVID-19 protective behaviours. In contrast, females, those with a lower education, those less likely to have experienced poverty since the beginning of lockdown; who perceived greater vulnerability to COVID-19, trusted government, and were more supportive of the behavioural measures were more likely to adhere to the restrictions of movement.

## Discussion

Our study suggests that South Africans were highly responsive to the COVID-19 pandemic with respondents generally reporting that they very often engaged in the protective behaviours (hand hygiene, wearing of face masks in public, and safe distancing outside the household) and often to very often adhered to government restriction on movement (no visiting of family, friends or neighbours; staying at home except for essential activities; not attending gatherings of more than 10 people). The high level of responsiveness may in part be explained by the current study being conducted during the second wave of the pandemic and thus covered a period of time when government engaged in widespread, high-level public communication on the pandemic, formed and engaged with a science advisory council, and implemented strict measures of movement to control the spread of COVID-19. The study also found that several social, economic, and psychological, factors were related to people’s responsiveness to the COVID-19 pandemic, with the association of specific social, economic, and psychological, economic and social factors differing for the adoption of preventive behaviour and adherence to government restrictions on movement.

The adoption of COVID-19 protective behaviours (hand hygiene, wearing of face masks in public, and safe distancing outside the household) was associated with the following social factors: population group, educational attainment and living circumstances. Age, gender and the financial impact (through the experience of poverty and loss of income) were not related to the adoption of COVID-19 protective behaviours. Adoption of these protective measures are informed by different factors. Those South Africans from the white population group, with a higher education, and living in uncrowded households were more likely to engage in COVID-19 protective behaviours indicating that access to resources influences the adoption of COVID-19 preventive behaviours. These results are also reflective of the racially based socioeconomic disparities in South Africa. Adherence to these non-pharmaceutical measures such as cleaning hands with soap and water, require access to essential services such as clean water [43]. Those who live in overcrowded living conditions, are most likely to live in informal settlements with inadequate infrastructure, such as limited water and sanitation, which makes adherence to protective measures such as wearing of face masks, hand hygiene, and social distancing difficult, if not impossible [44–46].

All the psychological factors (except for trust in government) were related to the adoption of COVID-19 protective behaviours. South Africans who perceived less vulnerability to contracting COVID-19; supported the measures; trusted the scientists; thought the measures by government were implemented fairly and fairly enforced by the police; felt more anxious, sad, hopeless, isolated, angry, or had trouble sleeping; and were more inclined to engage in coping behaviour, were more likely to adopt COVID-19 protective behaviours. These results are generally consistent with previous international research [16–18]. However, the finding that those who perceived less vulnerability to COVID-19 and those with more emotions such as anxiety, depression, and loneliness were associated with higher levels of engagement in COVID-19 protective behaviours is contrary to previous research [18, 36, 37, 47]. A possible explanation for this finding is that the perception of less vulnerability to contracting COVID-19 may reflect a greater confidence and understanding of their ability to reduce their exposure to the disease (self-efficacy) [16], while the association between emotions such as stress, anxiety, depression, and loneliness and the adoption of COVID-19 protective behaviours may be partly explained as a recognition of the seriousness of the disease and the need to curb its spread. While trust in the information provided by government was not associated with adoption of preventive behaviours, perceptions of the fairness of measures taken by government and the fairness in the enforcement measures were [20–24].

Adherence to government restrictions was also associated with several social and economic variables, namely gender, education, and financial impact, specifically the experience of poverty. Females, those with a lower education, and those who were less likely to have experienced poverty since the beginning of lockdown were more likely to adhere to government restrictions on movement. These findings resonate with other findings with regards to gender and education level [23, 46]. Since women are more likely to stay at home and care for their children because of traditional gender roles, and because child-care and learning institutions were closed during the pandemic, more women remained indoors rather than engaging in outside activities. Arguably, many in South Africa were not happy with lockdown measures regarding restrictions of movement, especially the businesses sector as temporary closure contributed to decreased turnover, and for some permanent closure and unemployment. Many big businesses, whose management are most likely to be highly educated and affluent, were averse to complying with restrictions of movement, and, thus, challenged government and took them to court on this matter. Our study also found that those who were less likely to have experienced poverty since the beginning of lockdown were more likely to adhere to government restrictions on movement. For poor people and informal traders in low-income communities in South Africa, the ability to earn a living is largely dependent on movement between different places or getting goods to local markets and trading centres [43] and therefore would have found it difficult to adhere to restrictions on movement.

However, compared to the adoption of COVID-19 protective behaviours, relatively few of the psychological factors were related to the adherence of government restrictions on movement. Those who perceived greater vulnerability to COVID-19, trusted government, and were more supportive of the behavioural measures were more likely to adhere to the restrictions of movement.

The study has several limitations. First the study used a cross-sectional design that cannot establish causal inferences. For example, it is possible that emotions such as anxiety and loneliness may have resulted from the adoption of COVID-19 protective behaviours such as staying at home. Another limitation relates to the use of a CATI survey with the results being biased to the cell phone population (e.g., higher percentage of women, with higher education and financial status than the national population). The results might also be limited to that section of the population from the telephone directories available in all nine provinces of South Africa who has access to a phone. Furthermore, while the measures in the study were based on a review of the literature and evaluated for face validity, some of the scales showed low to medium reliability and therefore the results should be interpreted with this in mind.

## Conclusions

The study provides useful information about the complex adoption of COVID-19 protective measures and adherence to government restrictions. Interventions to improve the adoption of COVID-19 preventive measures requires supporting those living in poor socioeconomic circumstances, promoting trust in the scientific evidence, and ensuring that the measures by government are perceived to be fairly implemented and fairly enforced by the police. On the other hand, because of the impact on livelihood, restrictions of movement should only be considered if necessary, and this will require trust and confidence in government and strategies to support those experiencing financial hardship. In the long-term, to cope with future pandemics, measures must be taken to address poverty and inequality in South Africa.

## Data Availability

The datasets used and/or analysed during the current study are available from the corresponding author on reasonable request.

## Footnotes

1 Participants could choose to identify their gender. As very few of the cases (less than 1%) identified as transgender or non-conforming gender, these could not be included in the statistical analysis.

2 The Apartheid regime classified people in South Africa in terms of their race as coloured, black, white, and Indian. These terms are used in this paper only for research and in support of redress purposes.
